# Antibodies for β2-Microglobulin and the Heavy Chains of HLA-E, HLA-F, and HLA-G Reflect the HLA-Variants on Activated Immune Cells and Phases of Disease Progression in Rheumatoid Arthritis Patients under Treatment

**DOI:** 10.3390/antib12020026

**Published:** 2023-03-31

**Authors:** Mepur H. Ravindranath, Narendranath M. Ravindranath, Carly J. Amato-Menker, Fatiha El Hilali, Senthamil R. Selvan, Edward J. Filippone, Luis Eduardo Morales-Buenrostro

**Affiliations:** 1 Department of Hematology and Oncology, Children’s Hospital, Los Angeles, CA 90027, USA; 2 Emeritus Research Scientist, Terasaki Foundation Laboratory, Santa Monica, CA 90064, USA; 3 Norris Dental Science Center, Herman Ostrow School of Dentistry, University of Southern California, Los Angeles, CA 90089, USA; 4 Department of Microbiology, Immunology, and Cell Biology, School of Medicine, West Virginia University, Morgantown, WV 26506, USA; 5 Medico-Surgical, Biomedicine and Infectiology Research Laboratory, The Faculty of Medicine and Pharmacy of Laayoune & Agadir, Ibnou Zohr University, Agadir 80000, Morocco; 6 Division of Immunology and Hematology Devices, OHT 7: Office of In Vitro Diagnostics, Office of Product Evaluation and Quality, Center for Devices and Radiological Health, Food and Drug Administration (FDA), Silver Spring, MD 20993, USA; 7 Division of Nephrology, Department of Medicine, Sidney Kimmel Medical College, Thomas Jefferson University, Philadelphia, PA 19145, USA; 8 Department of Nephrology and Mineral Metabolism, Institute of Nacional Medical Sciences and Nutrition Salvador Zubirán, Vasco de Quiroga 15, Sección XVI, Mexico City 14000, Mexico

**Keywords:** antibodies, IgM, IgG, β2-microglobulin, HLA-Ib, HLA-E, HLA-F, HLA-G, heavy chains, Face-1, Face-2, Face-3, Face-4, homodimers, heterodimers, immunosuppressive drugs

## Abstract

Rheumatoid arthritis (RA) is a progressive, inflammatory, autoimmune, symmetrical polyarticular arthritis. It is characterized by synovial infiltration and activation of several types of immune cells, culminating in their apoptosis and antibody generation against “altered” autoantigens. β2-microglobulin (β2m)-associated heavy chains (HCs) of HLA antigens, also known as closed conformers (Face-1), undergo “alteration” during activation of immune cells, resulting in β2m-free structural variants, including monomeric open conformers (Face-2) that are capable of dimerizing as either homodimers (Face-3) or as heterodimers (Face-4). β2m-free HCs uncover the cryptic epitopes that can elicit antibodies (Abs). We report here the levels of IgM and IgG Abs against both β2m and HCs of HLA-E, HLA-F, and HLA-G in 74 RA patients receiving immunosuppressive drugs. Anti-β2m IgM was present in 20 of 74 patients, whereas anti-β2m IgG was found in only 8 patients. Abs against β2m would be expected if Abs were generated against β2m-associated HLA HCs. The majority of patients were devoid of either anti-β2m IgM or IgG but had Abs against HCs of different HLA-Ib molecules. The paucity of anti-β2m Abs in this cohort of patients suggests that Abs were developed against β2m-free HLA HCs, such as Face-2, Face-3, and Face-4. While 63 of 68 patients had IgG Abs against anti-HLA-F HCs, 36 and 50 patients showed IgG Ab reactivity against HLA-E and anti-HLA-G HCs, respectively. Evidently, anti-HLA-F HC Abs are the most predominant anti-HLA-Ib HC IgG Abs in RA patients. The incidence and intensity of Abs against HLA-E, HLA-F, and HLA-G in the normal control group were much higher than those observed in RA patients. Evidently, the lower level of Abs in RA patients points to the impact of the immunosuppressive drugs on these patients. These results underscore the need for further studies to unravel the nature of HLA-F variants on activated immune cells and synoviocytes of RA patients.

## 1. Introduction

Chronic inflammatory, autoimmune, life-long debilitating diseases may predominantly involve activation of (i) the adaptive immune system (rheumatoid arthritis (RA), systemic lupus erythematosus (SLE), multiple sclerosis), (ii) the innate immune system (Crohn’s disease, ulcerative colitis), and (iii) a combination of both adaptive and innate immune systems (ankylosing spondylitis, psoriasis). The pathogenesis of these diseases is multifactorial due to complex interactions among genetic, environmental, and therapeutic factors. Therefore, it is difficult to identify a single or specific biomarker that could be used to define disease progression or identify an effective target for therapy. Developing personalized therapy for these diseases depends on distinguishing the shared and unshared events during their immunological and inflammatory progression. This investigation focuses on antibody responses to HLA structural variants in RA patients receiving a variety of immunomodulatory therapies.

For a better understanding of the diversity of the antibody responses, it is necessary to identify different stages of disease progression. RA in genetically susceptible individuals commences with asymptomatic synovial inflammation, followed by infiltration and activation of immune cells, proliferation, and antibody production against “altered” autoantigens, and culminates in hyperplasia of the joints with bone and cartilage degradation. Immunological events can be generally distinguished into three phases during disease progression [1,2,3,4]. Phase-I commences with infiltration of immune cells into the synovium, which is further accelerated during Phase-II [5,6]. Phase-II involves further infiltration; hyperactivation; and proliferation of T and B lymphocytes, neutrophils, macrophages, macrophage-like synoviocytes (MLSs), and fibroblast-like synoviocytes (FLSs) in the synovium. Furthermore, in this phase, pro-inflammatory cytokines are produced in the synovium, cartilage, and bone [7,8,9]. During this phase, abnormal B cell recognition leads to the production of autoantibodies. Some of those are generated against rheumatoid factor, anti-citrullinated protein, mutant citrullinated vimentin, and several other “altered” auto-proteins [6,10].

Phase-III demarcates deterioration of RA due to the induction of cell death, primarily by synovial apoptosis mediated by activated T cells and NK cells through the interaction of cell surface molecules of the TNF family, namely, the Fas antigen (CD95) and the Fas ligand (Fas-L). Apoptosis occurs in more than 50% of synoviocytes and T cells from synovial tissue and synovial fluid of RA patients [11]. While hyperactivation of immune cells promotes the proliferation and expression of cell surface “altered autoantigens”, culminating in their shedding, the occurrence of apoptosis-mediated cellular degeneracy also results in the release of cytoplasmic and cell-surface intact and “altered autoantigens” into the synovial fluid and then into circulation. The “altered autoproteins” expose the antigenic, cryptic domains of amino acids (epitopes) of the intact native proteins. Such exposure elicits the production of Abs. As usual, the first formed Abs against newly released “altered autoantigens” are IgM, followed by IgG Abs.

**Figure 1:**
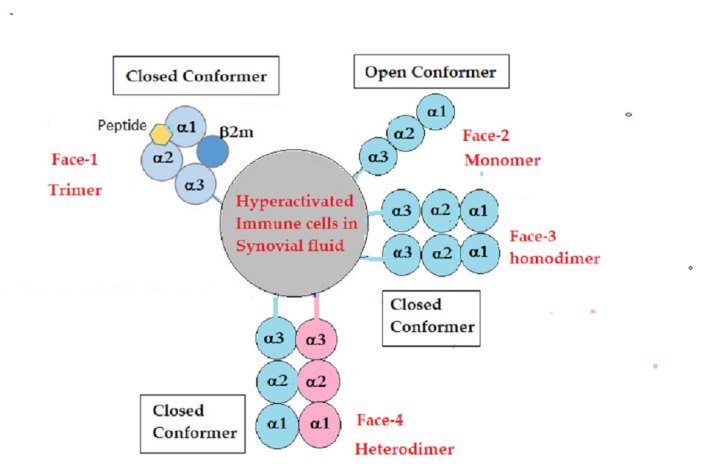
All immune cells, particularly lymphocytes, express cell-surface HLA class-I molecules as heterodimers consisting of HLA HC polypeptide and β2m, together with a peptide in the groove. They are considered trimers. They are also known as closed conformers (Face-1). Upon activation, these cells express monomeric variants of HLA class-I molecules and are referred to as open conformers (Face-2). The Face-2 versions of HLA-C, HLA-F, and HLA-G are also observed naturally on normal cells [14,15,16,17]. Face-2 is also observed in the monocytes of patients with spondylo-arthropathies and RA [18,19,20]. The monomeric version (Face-2) may dimerize with its own allele (homodimers or Face-3) or with other alleles of the same or different isomers (heterodimers or Face-4) [21,22,23,24].The figure shows different α-domains of the HC and different alleles in different colors.

The present investigation focuses on one such group of “altered auto-antigens”, namely, cell-surface HLA class-I. HLA class-I has diversified isomers, namely, HLA-A, HLA-B, HLA-C, HLA-E, HLA-F, and HLA-G. A pair of the same or different alleles of each of the six isomers is expressed on the cell surfaces of naïve and activated T and B lymphocytes, neutrophils, and macrophages. HLA-I molecules are expressed as heterodimers consisting of β2-microglobulin (β2m) non-covalently associated with the HLA heavy chain (HC). The intact β2m-associated HLA is referred to as a closed conformer [12] or Face-1 [13]. However, β2m-free HCs (Face-2) are also expressed on the cell surface during activation under different pathological conditions, such as inflammation, autoimmune diseases, and malignancy, as illustrated in Figure 1. Such Face-2 molecules may homodimerize (2-HCs of the same allele) to form Face-3 or heterodimerize (HCs of different alleles of same or different isomers) to form Face-4.

We hypothesize that when Face-1 molecules are shed into synovial and body fluids, both IgM and IgG can be formed against β2m, whereas when only β2m-free HCs (Face-2) are shed or recognized, the propensity for the appearance of anti-β2m can be virtually absent. Possibly, the IgM and IgG profiles of serum anti-HLA-HC Abs with or without anti-β2m Abs may reflect the different phases of disease progression and may serve as biomarkers for RA-progression-based therapies.

## 2. Material and Methods

### 2.1. Information on the Patient Cohort

The sera of 74 patients (57 females, 17 males) and sera of normal controls (26 males and 26 females) obtained from clinical facilities in Mexico were provided by Professor Dr. Luis Eduardo Morales-Buenrostro, Departamento de Nefrología y Metabolismo Mineral, Instituto Nacional de Ciencias Médicas y Nutrición Salvador Zubirán, Vasco de Quiroga 15, Sección XVI, Tlalpan, DF. 14000. MEXICO to Dr. Mikki Ozawa at TFL. The sera were collected after obtaining written informed consent approved by the Institutional Review Boards in Mexico by Dr. Morales-Buenrostro, and further approved for research by the ethics committee at Terasaki Foundation Laboratory, by the late Professor Paul Terasaki. All patients were seropositive for rheumatoid factor. All clinical details, including demographic characteristics of the patients studied and different combinational treatment regimens with the drugs received prior to the time of blood drawing and the information available and permissible by ethics committee are presented as a Supplemental Table S1 and discussed in appropriate context. All sera were kept at −20 °C before shipment and analyses of the sera.

### 2.2. Immunoregulatory Drugs Received by the Patient Cohort

The specific immunomodulatory therapies received by the cohort of patients are presented in Table 1. **Serum** IgM and IgG Abs were studied a month or more after administration of the drugs. Since the drugs are capable of suppressing antibody production, their mechanisms of action are summarized hereunder:

**Methotrexate [25,26]**: It inhibits dihydrofolate reductase, blocks the folic acid-dependent synthesis of purines and pyrimidines, inhibits T cell proliferation, diminishes macrophage recruitment and function, and promotes the release of adenosine, an anti-inflammatory mediator.

**Folic Acid [26]**: It reduces methotrexate’s adverse effects.

**Chloroquine [27]**: It inhibits MHC-II expression, antigen presentation, immune activation, and the production of pro-inflammatory cytokines. It interferes with Toll-like receptor 7 (TLR7) and TLR9 signaling pathways, and interferes with cyclic GMP-AMP synthase activity.

**Leflunomide (ARAVA) [28]**: It suppresses cell proliferation in activated lymphocytes; inhibits (i) dihydro-orotate dehydrogenase activity and protein tyrosine kinase activity in actively dividing cells, (ii) nuclear factor κB (NFκB) activation and NFκB-dependent reporter gene expression, (iii) oxygen free-radical generation, and (iv) immunoglobulin IgG and IgM production; and (v) lowers IL-1β and IL-2 levels.

**Prednisone [29]**: It inactivates NFκB, decreasing proinflammatory cytokine production; inhibits cyclooxygenase-2, adhesion molecules, and other inflammatory mediators, and importantly, suppresses IgM and IgG production.

**Azulfidine (Sulfasalazine) [30]**: It inhibits the release of IL-2 produced by T cells, and IL-1, IL-8, IL-12, and TFN-α produced by monocytes and macrophages; induces apoptosis of macrophages; inhibits the production of serum IgM and IgG; and suppresses infiltration of fibroblasts, neutrophils, and plasma cells.

**Azathioprine [31]**: It decreases leukocyte proliferation; promotes apoptosis of activated T cells and macrophages; reduces the expression of leukocyte adhesion molecules; and impairs NFkB signaling.

**Omeprazole [32]**: Omeprazole inhibits both basal and drug-stimulated gastric acid secretion in a dose-dependent fashion.

**ACEI (Angiotensin Convertase Enzyme Inhibitor) [33]**: It prevents the risk of hypertension and cerebrovascular disease, which are increased in RA patients.

**Table 1:** Specific immunomodulatory drug combinations administered to patients in different groups included methotrexate, folic acid, chloroquine, leflunomide, prednisone, azulfidine (Sulfasalazine), azathioprine, omeprazole and IECA (angiotensin convertase inhibitor). (+) indicates usage of the drug in the patients.

Chemotherapy Regimens for RA											Number of Patients with Serum IgM & IgG Antibodies Reacting to β2M, HLA-E/-F & G in Each Group					
	Methotrexate	Folic Acid	Chloroquine	ARAVA	Azulfidine	ACEI	Prednisone	Azathioprine	Omeprazole	Thyroid enzyme	Group 1 Table 2 14/16	Group2 Table 3 23/24	Group 3 Table 4 15/15	Group 4 Table 5 6/6	Group 5 Table 6 5/5	Group 6 Table 7 2/3
1	[+]	-	-	-	-	-	-	-	-	-		2				
2	[+]	[+]	-	-	-	-	-	-	-	-	2	2				
3	[+]	[+]	[+]	-	-	-	-	-	-	-	3	6	3		1	
4	[+]	[+]	[+]	[+]	-	-	-	-	-	-		2				
5	[+]	[+]	[+]	[+]	[+]	-	-	-	-	-	1					
6	[+]	[+]	[+]	-	-	-	[+]	-	-	-						1
7	[+]	[+]	[+]	-	[+]	-	-	-	-	-				1	1	
8	[+]	[+]	-	[+]	-	-	-	-	-	-		1		2		
9	[+]	[+]	-	[+]	[+]	-	-	-	-	-		1				
10	[+]	[+]	-	[+]	[+]	[+]	-	-	-	-	1					
11	[+]	[+]	-	[+]	-	-	[+]	-	-	-					1	
12	[+]	[+]	-	[+]	[+]	[+]	[+]	[+]	-	-		1				
13	[+]	[+]	-	-	[+]	-	-	-	-	-	1					
14	[+]	[+]	-	-	[+]	-	[+]	-	[+]	-			1			
15	[+]	[+]	-	-	-	-	[+]	[+]	-	-	1					
16	[+]	[+]	-	-	-	-	[+]	-	-	-					1	
17	[+]	[+]	-	-	-	-	[+]	-	[+]	-			1			
18	[+]	[+]	-	-	-	-	-	-	[+]	-			1			
19	[+]	[+]	-	-	-	[+]	-	-	-	-	1	1	1			
20	[+]	-	[+]	-	-	-	-	-	-	-	3	2		1		1
21	[+]	-	[+]	-	-	-	[+]	-	-	-	1					
22	[+]	-	[+]	[+]	-	-	-	-	-	-				1		
23	[+]	-	[+]	-	-	[+]	-	-	-	-		1				
24	[+]	-	-	-	[+]	-	-	-	-	-			1			
25	[+]	-	-	-	-	[+]	-	-	-	-		2				
26	[+]	-	-	-	-	-	[+]	-	-	-			2		1	
27	[+]	-	-	-	-	-	-	-	[+]	-		1				
28	-	-	[+]	-	-	-	-	-	-	-			2			
29	-	-	[+]	-	-	-	-	-	[+]	-			1			
30	-	-	[+]	[+]	[+]	-	[+]	-	[+]	-			1			
31	-	-	-	[+]	-	[+]	-	-	-	-		1	1			
32	-	-	-	[+]	-	[+]	-	-	[+]	[+]				1		

RA, Rheumatoid Arthritis; ACEI, Angiotensin Convertase Enzyme Inhibitor.

**Table 2:** Profiles of serum Abs in **Group 1** with 16 RA patients, one of whom had both RA and SLE. These patients did not have Abs against β2m, but had IgM against HCs of HLA-E (*n* = 13), HLA-F (*n* = 7), and HLA-G (*n* = 4) and IgG against HCs (HLA-E, *n* = 8; HLA-F, *n* = 14; and HLA-G, *n* = 12). The presence of Abs against HCs but not against β2m suggests that the immunogen was β2m-free HCs. Such a possibility can occur if the immune cells are hyperactivated and express β2m-free HCs, as Face-2, or even Face-3 and Face-4, as illustrated in Figure 1. When these β2m-free HCs are shed or released, the intact expression of β2m-associated HCs may remain unaffected on the cell surface. The shed β2m-free HCs may have elicited IgG.

	Patient ID	Other Complications	Treatment at Sampling	β2M	HLA-E HLA-F HLA-G Heavy Chains			β2M	HLA-E HLA-F HLA-G Heavy Chains		
				IgM	IgM	IgM	IgM	IgG	IgG	IgG	IgG
1	Alb-RA136F39		Met/Chlrqn/Predns	0	953	0	0	0	0	0	0
2	Alb-RA018M30		Met/Folic/Azulf	0	3721	1359	1747	0	0	0	1012
3	Alb-RA121F60		Met/Chlrqn/	0	771	0	0	0	0	585	0
4	Alb-RA112F39	Hypertns	Met/Folic/Azat/Predns	0	1172	553	0	0	0	585	0
5	Alb-RA 106F47		Met/Folic/ ACEI	0	2286	1011	0	0	0	518	777
6	Alb-RA113F77		Met/Chlrqn/	0	669	0	0	0	0	558	1227
7	Alb-RA082F51			0	923	0	0	0	0	888	862
8	Alb-RA045F32			0	651	0	0	0	867	1218	0
9	Alb-RA 125F43		Met/Folic	0	500	0	0	0	736	2099	1027
10	Alb-RA 034F48		Met/Chlrqn/ Folic/Azulf/	0	1695	0	622	0	987	774	1407
11	Alb-RA129F28		Met/Chlrqn/ Folic	0	2207	615	0	0	748	2330	1392
12	Alb-RA127M47		Folic/Met/ Chlrqn/	0	2780	0	0	0	607	653	2957
13	Alb-RA 095F64	Hypertns/diabet	Met/Folic	0	0	0	1498	0	0	753	779
14	Alb-RA098F64	Hypertns	Met/Chlrqn/Folic/	0	0	666	0	0	602	627	1317
15	Alb-RA012F57		Met/Chlrqn/	0	0	1258	0	0	811	1357	1306
16	Alb-RA 021F65	SLE/Hypertns diabetics	Azulf	0	1806	769	1035	0	1175	1242	1380

**Table 3:** Profile of serum Abs in **Group 2** with 24 RA patients, one of whom had both RA and SLE. Since these patients did not have IgM or Abs against β2m and only IgG against HCs of HLA-E (*n* = 13), HLA-F (*n* = 23), and HLA-G (*n* = 16), this group may represent a more advanced stage disease than that represented by Group 1. Shedding of HLA-F seems to have been more prevalent. The presence of Abs against HCs but not against β2m suggests that the immunogen should be β2m-free HCs, a possibility that can occur if the immune cells are hyperactivated and express β2m-free HCs, as Face-2 or even Face-3 and Face-4.

	Patient ID	Other Complications	Treatment at Sampling	β2M	HLA-E HLA-F HLA-G Heavy Chains			β2M	HLA-E HLA-F HLA-G Heavy Chains		
				IgM	IgM	IgM	IgM	IgG	IgG	IgG	IgG
1	Alb-RA032F54		Met/Chlrqn	0	0	0	0	0	0	520	0
2	Alb-RA039M31			0	0	0	0	0	0	682	0
3	Alb-RA031M71		Met	0	0	0	0	0	0	817	0
4	Alb-RA107F65		Met/chlrqn	0	0	0	0	0	0	1952	0
5	Alb-RA 096F41		Met/Chlrqn/Folic	0	0	0	0	0	0	647	0
6	Alb-RA126M26		Met/Chlrqn/Folic/	0	0	0	0	0	0	0	1416
7	Alb-RA102F61	Hyprtns/Thrmbss	Met/Folic/ ARAVA/Azulf/	0	0	0	0	0	0	668	547
8	Alb-RA094M66	Dyslipid	Met/Folic/	0	0	0	0	0	0	660	4138
9	Alb-RA037F51		Met/Chlrqn/Folic/ARAVA	0	0	0	0	0	0	1163	1763
10	Alb-RA049M66	SysVasc/ Hyprtns/ dslIpid/Ren Dis	Met/Folic	0	0	0	0	0	0	1294	946
11	Alb-RA002F35		Met/Folic/Arav	0	0	0	0	0	0	1587	872
12	Alb-RA115M40		Met/Omprz	0	0	0	0	0	512	645	0
13	Alb-RA076M60	Hyprtns/dyslipid	Met/Chlrqn/ ACEI	0	0	0	0	0	514	773	0
14	Alb-RA111F39	Hyprtns	Met/ACEI	0	0	0	0	0	758	849	0
15	Alb-RA058M32		Met/Folic/Azulf Predns/Omprz	0	0	0	0	0	540	559	508
16	Alb-RA020F26		Met	0	0	0	0	0	591	680	552
17	Alb-RA133F37		Met/Chlrqn/Folic/ARAVA	0	0	0	0	0	743	603	549
18	Alb-RA038F55	Hyprtns Hypothyr	Met/Chlrqn/Folic	0	0	0	0	0	524	957	809
19	Alb-RA103F72	Hyprtns	Met/Folic/ ACEI	0	0	0	0	0	512	661	1206
20	Alb-RA118F30		Met/Chlrqn/Folic	0	0	0	0	0	577	1148	510
21	Alb-RA099M76	Hyprtns	Met/AECI	0	0	0	0	0	561	1165	866
22	Alb-RA132F58	Hyprtns	Met/Chlrqn/Folic	0	0	0	0	0	651	1829	1528
23	Alb-RA135F55		Met/ Chlrqn/ Folic	0	0	0	0	0	1563	1084	1477
24	Alb-RA109F50	SLE/Hyprtns	Met/Folic/Azulf/ARAVA/Azat/ Predns/ ACEI	0	0	0	0	0	1133	719	934

**Table 4:** Profile of serum Abs in **Group 3** with 14 patients. These patients had IgM Abs against β2m. They had also developed IgM against HCs of HLA-E (*n* = 14), HLA-F (*n* = 7), and HLA-G (*n* = 8) and IgG against HCs (HLA-E, *n* = 5; HLA-F, *n* = 13; and HLA-G, *n* = 10). The presence of IgM against β2m indicates that the immunogen was an intact or β2m-associated HC of HLA-I (closed conformer or Face-1), as illustrated in Figure 1. The intact HLA were possibly released due to cell death. Shedding of HLA-F seems to have been more prevalent than other HLA-Ib molecules.

	Patient ID	Other Complications	Treatment at Sampling	β2M	HLA-E	HLA-F HLA-G		β2M	HLA-E	HLA-F HLA-G	
					Heavy Chains				Heavy Chains		
				IgM	IgM	IgM	IgM	IgG	IgG	IgG	IgG
1	Alb-RA 110F63		Met/Chlrqn/Folic	695	581	0	0	0	0	0	0
2	Alb-RA 114F61		Met/Folic/ IECA	2817	1910	0	0	0	0	510	0
3	Alb-RA 052F57		Met/Folic/ Predns/Omprz	3293	636	0	0	0	0	980	0
4	Alb-RA 092M56		Met/Predns	1134	618	0	0	0	0	1469	0
5	Alb-RA 051F44		Met/Chlrqn/Folic	1296	740	0	0	0	734	685	1106
6	Alb-RA 015F35	Hypothyrd	Azulf/ ARAVA/ Chlrqn/Predns/	850	2001	500	0	0	1020	766	1251
			Met/Chlrqn/								
7	Alb-RA 105F24		Folic/	593	4042	1209	2522	0	0	1022	829
8	Alb-RA 033F26		Met/Folic/Ompraz	603	632	1512	763	0	559	1239	1842
9	Alb-RA 014F73	Hypertens	Met/IECA	940	841	0	837	0	0	1207	1247
10	Alb-RA 100F21		Met/Chlrqn/ARAVA/Ompr	1008	1824	495	673	0	0	624	4518
11	Alb-RA 019F25		Chlrqn	1034	624	531	1016	0	0	1234	1093
12	Alb-RA 060F44		Met/Azulf	1317	1430	1541	3545	0	2076	1834	9985
13	Alb-RA 047F50		Chlrqn	1718	1117	973	1441	0	1104	1122	973
15	Alb-RA 138F55		Met/Folic/Azulf /Predns/	1728	786	0	2784	0	0	940	861

**Table 5:** Profile of serum Abs in **Group 4** with 6 patients. These patients have IgM but not IgG Abs against β2m (*n* = 6). These patients did not have IgM Abs against any of the HLA-Ib antigens. They had IgG Abs against HCs of HLA-E (*n* = 2), HLA-F (*n* = 6), and HLA-G (*n* = 5). The presence of IgM against β2m indicates that the immunogen could have been intact or β2m-associated HCs (closed conformers or Face-1) of HLA-Ia (HLA-A, HLA-B, and HLA-C) but not HLA-Ib. The intact HLA-Ia molecules were possibly released due to cell death. However, shedding of β2m-free HLA-F (Face-2, Face-3 and Face-4) seems to have been more prevalent than other β2m-free HLA-Ib molecules.

	Patient ID	Other Complications	Treatment at Sampling	β2M	HLA-E HLA-F HLA-G			β2M	HLA-E HLA-F HLA-G		
					Heavy Chains				Heavy Chains		
				IgM	IgM	IgM	IgM	IgG	IgG	IgG	IgG
1	Alb-RA 085F47	Dyslipid/diabet	Met/Chlrqn/ ARAVA/Statins	596	0	0	0	0	540	1626	1580
2	Alb-RA 048F41	Hpothyr/Hyprtns	ARAVA/Ompr/ Thyrd enzys/ IECA/B-block	638	0	0	0	0	0	1267	1464
3	Alb-RA 063M60		Met/Folic/ ARAVA	859	0	0	0	0	769	730	591
4	Alb-RA 124F29		Met/Chlrqn/ Folic/Azulf/	953	0	0	0	0	0	1289	683
5	Alb-RA 005M75		Met/Folic/ ARAVA	979	0	0	0	0	0	1602	1771
6	Alb-RA 043F24		Met/Chlrqn/ Folic/	1516	0	0	0	0	0	1142	0

**Table 6:** Profile of serum Abs in **Group 5** with 5 patients. These patients had no IgM but only IgG against β2m. Both IgM and IgG Abs against HCs of HLA-E/HLA-F/HLA-G were present. Anti-HLA-E and anti-HLA-F IgM were present in 4 patients and anti-HLA-G in 2 patients. Possibly, the intact HLA were released due to cell death.

	Patient ID	Other Complications	Treatment at Sampling	β2M	HLA-E HLA-F HLA-G Heavy Chains			β2M	HLA-E HLA-F HLA-G Heavy Chains		
				IgM	IgM	IgM	IgM	IgG	IgG	IgG	IgG
1	Alb-RA 027F37		Met/ ARAVA/ Predns	0	0	1816	0	578	1438	1356	2580
2	Alb-RA084M62		Met/Folic/ predns	0	763	637	0	825	822	1568	2829
3	Alb-RA 137F33		Met/Folic/azulf Chlrqn	0	1381	0	0	500	1086	1183	987
4	Alb-RA134F45		Met/Chlrqn/ Folic/	0	1607	840	654	861	2700	2763	3726
5	Alb-RA 088F34	Diabetes	Met/Predns	0	2237	1360	2799	754	1218	1525	3166

**Table 7:** Profile of serum Abs in **Group 6** with 3 patients. None of the patients had IgM Abs against β2m or HCs, as observed in **Group 2**. However, in contrast to **Group 2**, the patients in this group had IgG Abs against β2m and HCs.

	Patient ID	Other Complications	Treatment at Sampling	β2M	HLA-E HLA-F HLA-G Heavy Chains			β2M	HLA-E HLA-F HLA-G Heavy Chains		
				IgM	IgM	IgM	IgM	IgG	IgG	IgG	IgG
1	Alb-RA090F41		Met/Chlrqn/	0	0	0	0	752	1474	1577	1104
2	Alb-RA030M58			0	0	0	0	782	1812	3138	1531
3	Alb-RA097F47	Thrombosis	Met/Chlrqn/	0	0	0	0	573	778	0	0

### 2.3. Antigen Source

**Figure 2:**
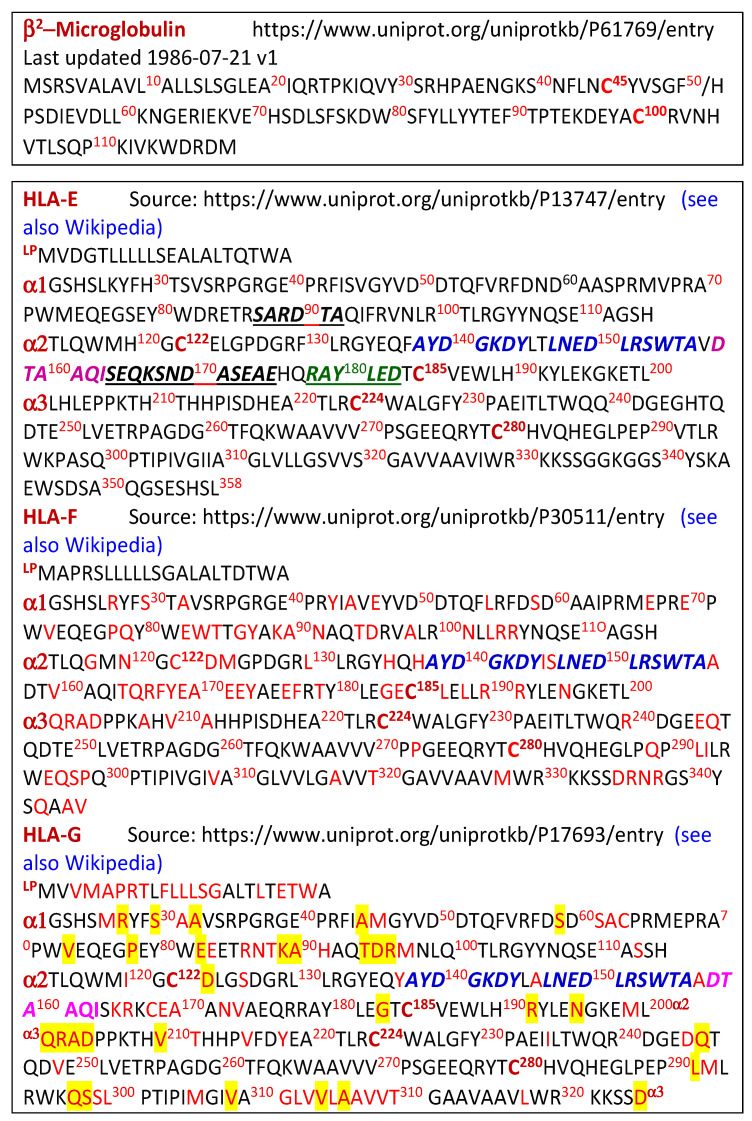
Amino acid sequences of β2m, and α1, α2, and α3 domains of the HCs of HLA-Ib isomers HLA-E, HLA-F, and HLA-G. Each sequence shown above from the β1 domain is the leader peptide (LP) of an HLA isomer. Letters in bold and italics refer to monospecific sequences for HLA-E (in black) that are specific for HLA-E and B*8201 (in green), shared with all HLA-Ia and Ib isomers (in blue), or shared with all HLA isomers except HLA-A and HLA-F (in red). Letters in red in HLA-F and HLA-G are the amino acids that differ from HLA-E. Red letters in yellow in HLA-G denote similarity to HLA-F. Note that HLA-E, HLA-F, and HLA-G have cysteine in the same position, as shown in the bold letter C in dark red with the position indicated in the superscript. The cysteine noted at positions 122, 185, 224, and 280 may facilitate homo- and heterodimerization of β2m-free HCs (Face-2), as shown in Figure 1.

Only HLA-Ib proteins were investigated in this study. Recombinant HLA-E, HLA-F, HLA-G, and β2m folded HCs (10 mg/mL in 2-[N-morpholino] ethanesulfonic acid [MES] buffer) were obtained from the Immune Monitoring Laboratory, Fred Hutchinson Cancer Research Center (University of Washington, Seattle, WA). Recombinant HCs of HLA-Ib alleles (HLA-E^R107^, HLA-F1, and HLA-G1) were folded and made available for coating microbeads. Figure 2 shows the amino acid sequences of the HCs of HLA-E^R^, HLA-F, HLA-G, and β2m used for coating the beads. All HLA-Ib alleles have only the extracellular domain without the leader peptide containing 21 amino acids, and no transmembrane or intracellular domains.

### 2.4. Immunoassay with Single Antigen Beads

To detect IgM and IgG reactivity to HCs of HLA-Ib isomers and β2m in the sera of RA patients and in normal males and females of the same ethnicity, a multiplex Luminex^®^-based immunoassay (One Lambda, Canoga Park, CA, USA) was used, as described earlier [34,35,36,37]. The recombinant HLA-E, HLA-F, and HLA-G HCs (10 mg/mL in MES buffer) and recombinant β2m (same concentration) were individually attached by a process of simple chemical coupling to differently fluorochromed 5.6 μm polystyrene microspheres internally dyed with infrared fluorophores. The sera were diluted 1:10 with phosphate-buffered saline (PBS, pH 7.2). Using dual-laser flow cytometry (Luminex^®^ xMAP^®^ multiplex technology), single antigen (AG) assays were carried out for data acquisition and analysis of anti-HLA-Ib Abs (39–40). For HLA-E, HLA-F, HLA-G, and β2m, positive (coated with IgG) and negative (coated with human or bovine albumin) controls were added separately. IgG and IgM screenings were performed using secondary anti-human IgG (One Lambda, cat. no. LS-AB2) and secondary anti-human IgM (Jackson ImmunoResearch Laboratories, Inc. West Grove, PA, cat. no. 709-116-073, USA), respectively. The secondary Ab was used at a dilution of 1/100. Data generated with Luminex Multiplex Flow Cytometry (LABScan^®^ 100, Thermo Fisher Scientific, Waltham, MA, USA) were analyzed using computer software, the protocol being the same as that reported earlier [33,34,35,36,37,38,39]. The mean and SD of the mean fluorescence intensity (MFI) for each allele were recorded.

Trimmed mean fluorescence values for the SAB reactions were obtained from the output (.csv was converted to .xls) file generated by the flow analyzer and were adjusted for blank and background signals using the formula below. To express the values of anti-HLA Abs at specified dilution, the sample specific fluorescent value (trimmed MFI) for each bead was taken into consideration. Four different kinds were obtained: (1) trimmed MFI with serum Abs; (2) MFI for HLA-coated beads added with PE-conjugated 2nd antibody only; (3) trimmed MFI of LABSCreen negative control (LSNC) sera with minimal or no HLA Abs; and (4) MFIs for the negative control beads (with PE-conjugated 2nd antibody only) used for LSNC. Normalized MFI is calculated as follows: {*MFI* (1)*—MFI* (2)}*—*{*(MFI of LSNC* (3)*—MFI* (4)}. The control beads included those coated with human IgG (positive control) and serum albumin (HSA/BSA) (negative control). For each analysis, at least 100 beads were counted. Mean and standard deviation of the MFI for each allele were recorded. All the data were stored and archived at TFL. Origin Graphics Software^®^ (OriginLab, Northampton, MA, USA.) was used to plot the data. Basic statistical analyses were carried out with Excel software. Only an MFI above 500 was considered positive at a 1/10 dilution of sera. MFI values <500 were recorded as “0” value in the tables.

## 3. Results

Abs were observed in the sera of 68 of 74 patients while receiving immunomodulatory drugs. These immunomodulatory drugs included methotrexate (63/74 patients) supplemented with folic acid (44/74), and chloroquine with methotrexate (14) or alone (3). In addition, several of the patients had also received combination therapy with leflunomide (ARAVA) and/or prednisone and/or azulfidine (sulfasalazine) and/or omeprazole and/or ACEI, as presented in Table 1. IgM and IgG Abs reacting to β2m and HCs of HLA-E, HLA-F, and HLA-G coated on microbeads were assessed for MFIs. These MFIs are considered to be semi-quantitative, as the MFI may vary with the lot of bead sets used, and each Labscreen bead set is admixed with both β2m-associated and β2m-free HCs [40,41,42].

### 3.1. Categorization of Sera Based on the Distributions of Anti-β2m and HCs Abs

**Figure 3:**
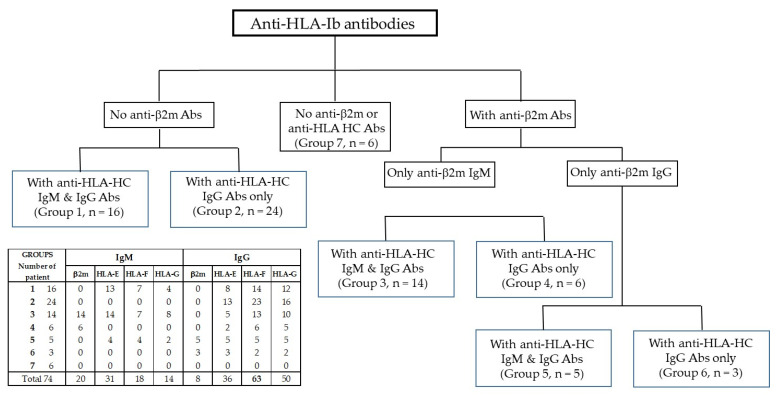
Profile of anti-HLA-Ib Abs in the sera of 74 RA patients. Sera can be broadly classified as those having anti-β2m Abs (*n* = 28, 20 with IgM, 8 with IgG) and those without anti-β2m Abs (*n* = 47). The table in the figure illustrates the detailed profile of IgM and IgG Abs against HCs of HLA-E, HLA-F, and HLA-G.

Based on MFIs obtained for IgM and IgG Abs, the patient sera were divided into categories based on the presence or absence of anti-β2m or anti-HC Abs, and the class of antibody (IgM or IgG). The major serum groups are shown in Figure 3, as follows:

Group 1. Sera (*n* = 16) with no anti-β2m IgM or IgG but with HLA-Ib HC IgM and IgG;

Group 2. Sera (*n* = 24) with no anti-β2m IgM or IgG but only with HLA-Ib HC IgG;

Group 3. Sera (*n* = 14) with anti-β2m IgM but not IgG;

Group 4. Sera (*n* = 6) with anti-β2m IgM but not IgG and with HLA-Ib HC IgG;

Group 5. Sera (*n* = 5) with only anti-β2m IgG together with HLA-Ib HC IgM and IgG;

Group 6. Sera (*n* = 3) with only anti-β2m IgG and HLA-Ib HC IgG;

Group 7. Sera (*n* = 6) with neither anti-β2m nor HLA-Ib HC Abs.

### 3.2. Group 1: Sera with No Anti-β2m IgM or IgG but with Anti-HLA-Ib HC IgM and IgG

The sera of 16 of 74 patients had neither anti-β2m IgM nor IgG but had both IgM and IgG against HCs of HLA-E, HLA-F, and HLA-G (Table 2). IgM Abs formed against HLA-E were present in more patients (*n* = 13) compared to those formed against HLA-F (*n* = 7) and HLA-G (*n* = 4). In contrast, IgG against HCs of HLA-F (*n* = 14) and HLA-G (*N* = 12) were present in more patients compared to those against HC HLA-E (*n* = 8). The presence of Abs against only HCs of HLA-Ib loci and not against β2m suggests that the immunogen may have been β2m-free HLA variants (Face-2, Face-3, and Face-4), instead of an intact HLA (Face-1).

### 3.3. Group 2: Sera with No Anti-β2m IgM or IgG but Only with Anti-HLA-Ib HC IgG

Sera of 24 of 74 patients had neither anti-β2m IgM nor IgG, but had only IgG against HCs of HLA-E (*n* = 13), HLA-F (*n* = 23), and/or HLA-G (*n* = 16) (Table 3). Once again, the prevalence of IgG against HCs of HLA-Ib loci without any IgM or IgG against β2m strongly suggests that the immunogen may have been β2m-free HCs (Face-2) of the HLA, rather than intact HLA molecules (Face-1).

### 3.4. Group 3: Sera with Anti-β2m IgM Only but with Anti-HLA-Ib HC IgM and IgG

The sera of 14 patients had only anti-β2m IgM but not anti-β2m IgG. However, both IgM and IgG Abs against the HCs of HLA-E (IgM *n* = 14, IgG *n* = 5), HLA-F (IgM *n* = 7, IgG *n* = 13), and HLA-G (IgM 8, IgG *n* = 10) were detectable (Table 4). The presence of high MFIs of IgM against β2m and HLA-E suggests the release or shedding of intact HLA-E molecules (Face-1) from selected immune cells, possibly due to cell death. The prevalences of IgM Abs against β2m and HCs indicate not only the commencement of cell death but also Phase-III of immunological progression (Phase IIIa). The high prevalence of anti-HLA-F HC IgG in 14 of 15 patients suggests that one or more of the activated immune cell types may express β2m-free HLA-F variants (Face-2, Face-3, and Face-4).

### 3.5. Group 4: Sera with Anti-β2m IgM Only without Anti-HLA-Ib HC IgM but with IgG

The sera of six patients had anti-β2m IgM only and not anti-β2m IgG. No IgM Abs against the HCs of HLA-Ib were noted. IgG Abs against HLA-E (IgG *n* = 2), HLA-F (IgG *n* = 6), and HLA-G (IgG *n* = 5) were detectable in patients (Table 5). The presence of IgM against β2m without any IgM against HCs of HLA-Ib suggests that the Abs against β2m may have been due to the release or shedding of β2m from the cell surface as intact HLA-Ia molecules (Face-1), possibly due to cell death in Phase III. The presence of IgG Abs against HCs of HLA-Ib without anti-β2m IgG suggests that the immunogen may have been from β2m-free HCs (Face-2) of the HLA-Ib. The absence of IgM Abs against HCs in the presence of IgG Abs against HCs supports the contention that the patients may have represented a phase subsequent to Phase-IIIa (Phase-IIIb).

### 3.6. Group 5: Sera with Anti-β2m IgG but Not IgM Together with HLA-Ib HC IgM and IgG

Sera of five patients had only anti-β2m IgG but not IgM, and had both IgM and IgG against the HCs of HLA-E (IgM *n* = 4, IgG *n* = 5), HLA-F (IgM *n* = 4, IgG *n* = 5), and HLA-G (IgM *n* = 2, IgG *n* = 5) (Table 6). The presence of anti-β2m IgG and the high MFIs of IgG and IgM against HLA-E, HLA-F, and HLA-G suggest that they could have been a consequence of the release or shedding of intact HLA-Ib molecules (Face-1) from several types of immune cells, possibly due to their cell death. The MFIs of IgG Abs against HLA-E, HLA-F, and HLA-G were higher than for other groups of patients. Since the patients had no IgM but only IgG against β2m, and they had both IgM and IgG against HCs of HLA-Ib, they may represent a phase subsequent to Phase-IIIb (Phase-IIIc).

### 3.7. Group 6: Sera with Anti-β2m IgG but Not IgM and with HLA-Ib HC IgG but Not IgM

Sera of three patients had anti-β2m IgG but no IgM and had IgG but no IgM Abs against the HCs of HLA-E, HLA-F, and HLA-G (Table 7). It appears that this group reflects a more advanced disease stage than the one represented by Groups 4 and 5, as IgM was no longer detectable. These patients may represent a phase subsequent to Phase-IIIc (Phase-IIId).

### 3.8. Group 7: Sera with Neither Anti-β2m Nor HLA-Ib HC Abs

In this group of sera (*n* = 6), neither anti-β2m nor anti-HLA-Ib HC IgM or IgG Abs were observed (Table 8). These patients received almost the same therapy as those in other groups. Could it be possible that these patients reflect those receiving therapies for longer durations? Or could the immunosuppressive therapies have been more efficacious in this group of patients?

### 3.9. High Levels of Anti-HLA-Ib IgM and IgG Abs in Normal Males and Females of the Same Ethnicity as the RA Patients

For the purpose of comparison of the profiles of anti-HLA-Ib Abs with the normal controls of the same ethnicity of the patients, we examined serum IgM and IgG Abs of normal males and females using Luminex multiplex flow cytometry. The profiles of Abs in normal individuals against HLA-E, HLA-F, and HLA-G are presented in Table 9. It is important to note that the incidence and strength (MFI) of anti-HLA-E, anti-HLA-F, and HLA-G IgM and IgG Abs were consistently high in almost all patients, in contrast to the patient cohort.

When comparing the Ab-profile normal cohort with patients, it can be noted that none of the patients in Group1 (Table 2), Group2 (Table 3), Group4 (Table 5), or Group6 (Table 7) had both IgM and IgG against all three HLA-Ib molecules. Both IgM and IgG against all three HLA-Ib molecules were observed in 3 of the 15 patients in Group3 (Table 4, Alb-RA 033F26, Alb-RA 060F44, and Alb-RA 47F50) and 2 of the 5 patients in Group5 (Table 6, Alb-RA 134F454 and Alb-RA 088F34).

## 4. Discussion

### 4.1. Anti-HLA-F IgG without Anti-β2m-IgG Is Most Prevalent in RA Patients

The highest prevalence (63/68) of anti-HLA-F IgG and the lowest prevalence (8/69) of anti-β2m IgG suggest that the anti-HLA-F IgG may not be formed against intact HLA-F molecules or Face-1 HLA-F, but rather appears to be formed against β2m-free HLA-F (Face-2, Face-3, or Face-4 of HLA-F). Based on our findings [41,42] on the categories of monoclonal Abs (mAbs) generated after immunizing HLA-E HCs in mice, it was clarified that the Abs formed could be HLA-E-monospecific (e.g., mAbs TFL-033, TFL-034, and TFL-145) or polyreactive to many or all HLAs, as illustrated in Table 10. Highly polyreactive mAbs (e.g., TFL-006 and TFL-007) raised against Face-2 of HLA-E react to HLA-A, HLA-B, HLA-C, HLA-E, HLA-F, and HLA-G. These Abs recognized the most shared amino acid sequences (AYDGKDY and LNEDLRSWTA) of other HLA-I molecules, as shown in blue letters in Figure 2.

However, the higher prevalence of anti-HLA-F IgG compared to those of HLA-E and HLA-G suggests that the Abs may have been formed specifically against HLA-F, such as the β2m domain sequence ^164^TQRFYEAEEY^173^, shown in red in Figure 2. In support of this contention, we can observe the following patterns.

(1) Anti-HLA-F IgG was observed in 64 of 69 patients. Only anti-HLA-F IgG was observed in 12 of 69 patients, indicating that these IgG Abs were specific for HLA-F. Thus, only anti-HLA-F IgG was observed in 2 of 16 Group 1 patients (Table 2), 5 of 24 patients in Group 2 (Table 3), 4 of 15 Group 3 patients (Table 4), and 1 of 6 Group 4 patients (Table 5).
(2) The MFI of anti-HLA-F IgG was higher than the MFI of anti-HLA-E or anti-HLA-G in 7 of 16 patients in Group 1 (Table 2), 17 of 24 patients in Group 2 (Table 3), 8 of 15 patients in Group 3 (Table 4), 3 of 6 patients in Group 4 (Table 5), 1 of 5 patients in Group 5 (Table 6), and 2 of 3 patients in Group 6 (Table 7).

These findings suggest that the IgG Abs may formed against sequences of HLA-F shared with other HLA HCs. Formation of HLA-F IgG Abs in the absence of anti-β2m IgG strongly favors the view that these Abs are generated against β2m-free HLA-F variants (Face-2, Face-3, and/or Face-4) rather than from the β2m-associated HLA-F (Face-1).

A primary alteration in activated cells involves the formation of a persistent HLA variant without β2m, referred to as open conformer [12] or Face-2 [13]. An earlier study performed [43] on a murine HLA (H-2D^b^) found that β2m is not required for cell surface expression of HLA, shattering the previously held dogma [44] that the HCs of HLA-I can be conformationally stable on the cell surface only as heterodimers with β2m. Using the β2m-free, HLA-HC-specific mAb LA45, Schnabl et al. [45] first reported the presence of the Face-2 variant on the cell surfaces of both in vitro and in vivo activated human T lymphocytes. Several monoclonal Abs were developed against Face-2, which include LA45, L31, TFL-006, and TFL-007. Madrigal et al. [46] observed the expression of the LA45 epitope on lectin-activated T cells. After phytohemagglutinin (PHA) activation of T cells isolated from 12 healthy individuals, the cell surface became distinctly positive with LA45. Demaria et al. [47] observed the formation of the Face-2 variants on human peripheral blood T cells stimulated with phorbol myristate acetate (PMA), anti-CD3 Ab, or PHA. Indeed, all of the HLA-I isomers are expressed as Face-2 variants on activated cells. Using mAb L31, HLA-C was first observed to express Face-2 naturally on a subpopulation of “normal cells”; however, the density of Face-2 was higher on 20 different kinds of EBV-transformed B lymphoid cell lines, expressing CW1 through CW8 [48] and on activated T cells of transgenic mice [49]. Similarly, using LA45 and other mAbs, the presence of HLA-F Face-2 variants was documented on activated T lymphocytes [50,51,52]. The presence of HLA-G Face-2 was observed on trophoblast cells in first-trimester human placental tissues with a HLA-G Face-2-specific mAb MEM-G/01 [53]. Face-2 variants were also observed on several human cancers (neuroblastoma cell lines IMR-32 and LAN-1) [14] and on colon [54], breast, ovarian, renal, and bladder carcinoma and human melanoma cell lines [15,16]). Studies on Face-2 of HLA-G revealed that the exposed amino acids may once again get masked due to homodimerization of Face-2 molecules to become Face-3 or due to heterodimerization of Face-2 of different alleles to become Face-4 [17,55,56].

Similar findings are observed when studying HLA-Ia loci. Face-2 of HLA-B27 in thymic epithelial cells and a subpopulation of peripheral blood lymphocytes of B27-transgenic mice contributed to the development of arthritis [57,58]. Indeed, Bix and Raulet [59] established that functionally conformed, free class-I HCs (Face-2) existed on the surfaces of β2m-negative cells. Face-2 of HLA-B27 exposes cysteine at position 67 in the extracellular β1-domain, which is otherwise masked by β2m.

Most importantly, the increased expression of Face-2 was observed not only with HLA-B27, but also with other HLA-Ia loci (HLA-B and HLA-C) on monocytes of patients with spondylo-arthropathies (SA) and RA [18,19,20,60,61,62]. Ding et al. [19] observed that the Face-2 expression was more “strongly associated with synovial fluid (SF) cells than peripheral blood (PB) cells and closely associated with SA disease activity” (page 8). However, they found no correlation between free HCs (FHC) and HLA-B27 expression in SA patients, as has been reported earlier by others [60,61,62]. This is possibly due to the presence of free HCs of other HLA-I isomers, including HLA-A, HLA-B, HLA-C, HLA-E, HLA-F, and HLA-G. Therefore, they [19] prudently pointed out “the increased relative FHC expression that (they) observed on the surface of SA monocytes may not have all been ascribed to HLA-B27 alone” (page 9).

Allen et al. [21] documented the formation of Cys^67^-dependent HC homodimers of Face-3 in β2m-free HLA-B27. Subsequently, exposed Cys^101^ and Cys^164^ in β2m-free HCs were also shown to participate in B27 homodimer (Face-3) formation [22]. Santos et al. [23] reported the induction of HLA-I Face-3 after activation of dendritic cells. HLA-B27 Face-2 and Face-3 were observed to form strong ligands for leukocyte Ig-Like receptors (LILRB2) [63] and KIR3DL2 [24]. Such binding of KIR and LILR family receptors with Face-3 and Face-4 of HLA can down-regulate T cell receptor-mediated T cell activation and inhibit NK cell production of INF-γ to suppress further activation of NK and T cells in SA and RA [19].

Importantly, apoptosis of immune and synovial cells results in the shedding of different HLA variants (Face-1, Face-2, Face-3, and Face-4) into synovial fluid and then into circulation. These HLA variants, particularly Face-2, may expose amino acid sequences or epitopes previously masked by β2-microglobulin. Upon exposure, these cryptic epitopes become immunogenic and can elicit Abs [35,64]. Some of these exposed cryptic sequences are shared among different HLA-I alleles, such as AYDGKDY among all the isomers of classical HLA-Ia (HLA-A, -B, and -C) and non-classical HLA-Ib (HLA-E, -F, and –G) loci. After immunizing mice with Face-2 of HLA-E, some of the resulting monoclonal Abs (TFL-006 and TFL-007) recognized the shared epitopes of all loci of HLA-Ia and HLA-Ib [41,42]. However, when Face-1 HLA-I are shed, the Abs may form not only against HCs, but also against β2m, which is not present in the other HLA-variants (Face-2, Face-3, and Face-4).

### 4.2. Unique Structural Variants of HLA-F on Activated Immune Cells in RA

**Figure 4:**
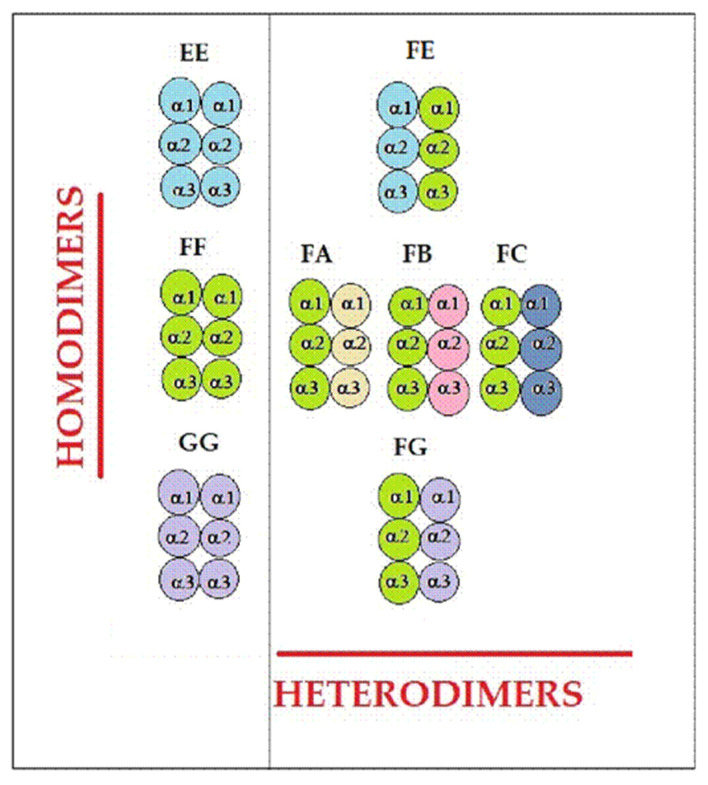
Diagrammatic illustration of HLA-E, HLA-F, and HLA-G homodimers and possible formation of HLA-F heterodimers with HLA-A, HLA-B, HLA-C, HLA-E, and HLA-F HCs. Different colors of the domain refer to differences in the alleles.

HLA-F is frequently expressed, without β2m (Face-2), on activated lymphocytes [65] and on proliferating lymphoid and monocyte cells [66]. DNA microarray analysis further revealed an abnormal network associated with HLA-F in bone marrow cells from patients with RA [67]. Furthermore, using comprehensive gene-expression meta-analysis, it was documented that the expression of the HLA-F gene is significantly upregulated in PBMCs of clinical RA patients [68,69]. It is well known that among the HLA-I (Face-2), HLA-F is more stable, and it has a propensity to bind to NK cell Ig-like receptor KIR3DSI [66] and different alleles of other HLA-I HCs or Face-2 molecules to form homodimers (Face-3) or heterodimers (Face-4), as diagrammatically illustrated in Figure 4. The presence of HLA-F HCs or Face-2, and Face-3, and Face-4 molecules may possibly account for the production anti-HLA-F IgG without the presence of β2m Abs in RA patients.

If Abs were formed against intact or β2m-associated HLA HCs (Face-1), one can expect Abs against β2m. However, only 15 of 61 RA patients’ sera had only IgM anti-β2m Abs, and 8 had only anti-β2m IgG Abs. The rest of the 46 patients were devoid of either anti-β2m IgM or IgG. The paucity of anti-β2m Abs suggested that Abs would have been developed against β2m-free HLA HCs, as illustrated in Figure 4. With these unique variants of HLA, which are often reported on the cell surfaces of activated immune cells, it is logical to expect Abs against HCs without anti-β2m.

### 4.3. IgM and IgG HLA-Ib Abs with and without Anti-β2m Abs May Reflect the Phases of Immunological Progression during Immunosuppressive Therapies

While intact or β2m-associated HLA HCs (Face-1) are prevalent in all immune and non-immune cells, β2m-free HLA HCs (Face-2) are found predominantly after the activation of immune cells. It is well known that in RA patients, several immune cells are hyper-activated, suggesting the prevalence of the Face-2 variant of HLA. Face-2 homo- or heterodimerization forms Face-3 or Face-4 variants, as shown in Figure 4. Abs formed against HCs in the absence of anti-β2m-IgM or IgG mark the early phases of immunological progression of RA, characterized by the infiltration of activated immune cells into the synovium. Cell death, mostly by apoptosis, is the most important event taking place in the final phase of RA. The release of β2m from intact HLA (Face-1) can be expected, which may lead to the formation of anti-β2m-IgM, followed by anti-β2m-IgG. Therefore, the patients (n = 40) with sera devoid of anti-β2m-IgM or IgG may represent those in early stages of RA, and the 15 patients sera with anti-β2m-IgM or anti-β2m IgG may represent advanced phases of immunological progression of RA, as summarized in Table 11.

Despite the defined immunological progression of patients with RA, it is important to note that almost all the patients in our cohort were receiving a variety of immunosuppressive drugs. Most importantly, several drugs interfere with the natural immunological progression of RA, as presented earlier in Material and Methods. Since these drugs are used in combination with other drugs, it is not possible to create categories of antibody response in conjunction with the specific kind of drug received. However, the immunological responses can be classified broadly as follows:

(1) Drugs that suppress cell proliferation of activated immune cells. Methotrexate, leflunomide, and azathioprine belong to this category.
(2) Drugs that inhibit IgM and IgG production. Leflunomide, prednisone, and azulfidine (sulfasalazine) belong to this category.
(3) Drugs that promote apoptosis of activated human T cells and immune cells. Azulfidine (sulfasalazine) and azathioprine belong to this category.
(4) Most of the drugs listed in Table 1 suppress pro-inflammatory cytokines.

Therefore, our results on anti-HLA HC Abs not only reflect immunological progression of the disease but also the impacts of the drug treatments the patients received. For example, Azulfidine and azathioprine promote the apoptosis of activated T cells and macrophages. Consequently, intact HLA molecules with β2m may be released into the circulation, which would have promoted production of anti-β2m IgM, as in patients Alb-RA-051F35 and 017F64 (Table 4). These anti-β2m IgM may be azulfidine-dependent. However, the rest of the 5 RA patients who had anti-β2m IgM Abs (Table 4) were not treated with drugs promoting apoptosis. Therefore, they may represent the last phase of the progression of natural events, characterized by cell death (apoptosis) of immune cells in the synovium.

The sera without the presence of anti-β2m-IgM or IgG in 47 patients could have been a consequence of immunosuppressive treatments. A higher prevalence of anti-HLA-F HC Abs was found compared to other HLA-Ib loci. Once again, the high prevalence of anti-HLA-F IgG (64/69), in contrast to the low prevalence (8/76) of anti-β2m IgG, suggests that HLA-F variants (Face-2, Face-3, and Face-4; Figure 4) are indeed the major immunogenic antigens in RA patients. This highlights the need to characterize the expression of variants of HLA-F in immune and in non-immune cells of RA patients, which may shed a brighter light on the immunodynamics of HLA-F specifically, and HLA-Ib Abs in general, in RA patients.

## 5. HLA-Ib Antibody Profiles in the Normal Control Group

Naturally occurring IgM and IgG antibodies in normal and healthy volunteers were studied earlier [63]. Comparatively, the incidences of MFI of IgM and IgG antibodies against HLA-E, HLA-F, and HLA-G in normal males and females are considerably higher than those observed in RA patients. Evidently, the antibodies levels stay lower in RA patients, confirming the impact of the immunosuppressive drugs received by the patients. The most striking feature is that only 5 females of the 69 patients examined had both IgM and IgG serum Abs at comparable level to the normal cohort. It is possible they were yet to be impacted by the immunosuppressive drugs. Therefore, studying both IgM and IgG antibodies against HLA-Ib before and during treatment of immunosuppressive drugs, at different doses, may reveal progressive and possibly tolerable drug mediated immunosuppression. It is under these circumstances that the high prevalences of anti-HLA-F IgG antibodies in contrast to those of anti-HLA-E IgG and anti-HLA-G IgG in the majority of the RA patients is noteworthy. These findings strongly favor monitoring anti-HLA-F IgG in RA patients while they receive treatment protocols and may serve a biomarker to regulate dosage and combination drugs during the course of the disease until a total loss of antibody response, as observed in Group 7.

## 6. Limitations of This Investigation

Since this investigation was carried out on sera obtained from a large cohort of patients visiting different clinical centers in Mexico, the following detailed information could not be obtained: (i) the dosages received for individual drugs for each patient, (ii) the time interval between date of sera collection and the duration of initiation of the drug administration prior to sera collection, (iii) the disease severity (DAS28, CDAI, etc.), and (iv) the disease duration after serum collection. It would be worthwhile if every patient were to be tested for antibodies against not only rheumatoid factor, but also cyclic citrullinated peptide to assess correlations between these classical seropositive biomarkers and anti-HLA-Ib antibodies.

In addition, analysis of serum anti-HLA-Ia (HLA-A, HLA-B, and HLA-C) IgM and IgG antibodies would be valuable, since HLA-Ia and HLA-Ib share several amino acid sequences (such as ^141^AYDGKDY^147^ and ^152^EDLARSWTA^159^). There is a compelling need to undertake a detailed investigation on the analysis of IgM and IgG antibodies reacting to all isomers of HLA-Ia and HLA-Ib, rheumatoid factor, and cyclic citrullinated peptide during the well-defined course of RA disease progression, to validate the hypothesis proposed in this investigation.

## 7. Summary

This investigation reported the differences in the strengths (MFI) of IgM and IgG Abs against β2m and HCs of HLA-E, HLA-F, and HLA-G in 74 RA patients. Twenty-nine of seventy-six patients’ sera had anti-β2m Abs. Of these, 21 had anti-β2m IgM, and 8 had only anti-β2m IgG. One can reasonably expect that the Abs against β2m were generated against intact or β2m-associated HLA HCs (Face-1). The vast majority of the remaining patients were devoid of either anti-β2m IgM or IgG but had Abs against HCs of different HLA-Ib molecules. This suggests that Abs against β2m-free HLA HCs, such as Face-2, Face-3, and Face-4, have been developed. Most strikingly, anti-HLA-F IgG Abs were observed in 92.7% of RA patients examined. Both the nature and the immunogenicity of variants of HLA-F on immune and non-immune cells of RA patients deserve further in-depth correlative investigation.

## Figures and Tables

**Table 8:** Profile of serum Abs in Group 7 with 6 patients. Most unusually, both IgM and IgG Abs against β2m and HLA-Ib HCs were totally absent, possibly due to the durations or types of therapies the patients received.

	Patient ID	Other Complications	Treatment at Sampling	β2M	HLA-E HLA-F HLA-G Heavy Chains			β2M	HLA-E HLA-F HLA-G Heavy Chains		
				IgM	IgM	IgM	IgM	IgG	IgG	IgG	IgG
1	Alb-RA091F30		Met	0	0	0	0	0	0	0	0
2	Alb-RA024F54	Hpothyr	Met/Thyoid Enz	0	0	0	0	0	0	0	0
3	Alb-RA 065F59	Hpertns/Diabet	Met/Chlrqn/Folic	0	0	0	0	0	0	0	0
4	Alb-RA104F22		Met/Chlrqn/ Predns	0	0	0	0	0	0	0	0
5	Alb-RA130F51		Met/Chlrqn/ Folic/Omprz	0	0	0	0	0	0	0	0
6	Alb-RA074F37		Met/Chlrqn/ Folic/Azul	0	0	0	0	0	0	0	0

**Table 9:** Profiles of IgM and IgG antibodies formed against HCs of HLA-E, HLA-F, and HLA-G in normal male and female Mexicans, representing the control cohort.

Males	HLA-E		HLA-F		HLA-G		Females	HLA-E		HLA-F		HLA-G	
	IgM	IgG	IgM	IgG	IgM	IgG		IgM	IgG	IgM	IgG	IgM	IgG
AT-252	6524	0	2334	1441	3103	2585	AT-63	7369	704	5729	1396	7493	1995
AT-126	3172	884	2139	1506	6177	4761	AT-212	6827	653	3236	947	4835	1701
AT-48	6157	1106	1743	2086	4906	4228	AT-56	4972	728	781	2086	2209	2397
AT-364	3953	730	1146	1511	1989	2331	AT-343	4739	915	1722	1452	3086	1974
AT-400	3948	632	1305	1002	2616	2464	AT-372	4280	1075	1778	2199	5917	2895
AT-449	2923	886	749	2069	1969	3717	AT-1	3510	936	1166	1873	3738	2156
AT-154	2817	803	1621	1728	1988	2995	AT-323	3255	897	1534	1315	3715	2423
AT-222	2230	1154	923	1387	1120	3765	AT-253	3130	717	1841	1430	4206	3511
AT-229	2144	1798	665	2002	1260	3578	AT-362	2377	0	1492	1105	3773	1915
AT-359	1804	1143	872	1982	2170	3216	AT-38	2666	1313	1761	1823	2486	3427
AT-393	1764	1115	919	1455	2213	3138	AT-18	2448	1071	1994	1466	3690	2526
AT-304	1623	0	980	1428	1403	2932	AT-374	2212	789	685	1555	1483	3112
AT-54	1490	1849	1469	2186	1762	3413	AT-109	2128	1510	1004	2612	1941	3882
AT-277	1360	1134	769	2263	1556	2487	AT-330	2091	928	574	1927	2192	2923
AT-82	1279	1028	875	1649	1259	3051	AT-290	2000	2203	1060	2333	2521	3849
AT-88	1227	834	0	1693	987	4700	AT-150	1887	533	1347	1127	3287	3554
AT-239	1216	652	784	1402	2019	3236	AT-300	1866	1018	961	1800	2815	4152
AT-338	1118	1141	0	1788	1009	3430	AT-191	1663	1728	1476	3156	2788	2995
AT-438	1049	877	0	2402	1005	2818	AT-78	1621	685	823	1248	1580	1637
AT-392	741	1314	1219	1963	1379	3477	AT-89	1483	670	734	1404	2284	3811
AT-386	960	1121	0	1945	1038	3220	AT-200	1476	1042	577	1875	1362	2933
AT-133	822	1738	979	3049	624	4756	AT-181	1402	623	1052	1350	1938	1775
AT-242	735	1206	554	1600	1680	2626	AT-70	1302	790	0	1832	1225	2514
AT-145	667	1258	0	1626	1263	2405	AT-112	1137	0	995	935	1780	1750
AT-219	0	639	0	2249	778	2321	AT-140	802	1007	516	2083	1555	3042
AT-354	0	1437	0	2778	641	3670	AT-412	802	994	0	1794	1823	2495
Mean	1989	1018	848	1853	1843	3281	Mean	2671	905	1340	1697	2912	2744
Median	1425	1111	874	1758	1479	3218	Median	2110	906	1056	1675	2504	2711
SD	1650	446	673	460	1258.4	729.99	SD	1712	454	1123	520	1433	767

**Table 10:** Diversity of the monoclonal Abs generated after immunizing mice with recombinant HC of HLA-E (HLA-E^G107^ or HLA-E^R107^) revealed monospecific (Category 1) and different categories of polyreactive mAbs, which included HLA-Ib-specific and HLA-Ia polyreactive mAbs. [−]; Negative or not reactive; [+] Positive or reactive.

Categories of mAbs	HLA-Ia			HLA-Ib		
	HLA-A	HLA-B	HLA-C	HLA-E	HLA-F	HLA-G
Category 1	[−]	[−]	[−]	[+]	[−]	[−]
Category 2	[−]	[−]	[−]	[+]	[+]	[−]
Category 3	[−]	[−]	[−]	[+]	[−]	[+]
Category 4	[−]	[−]	[−]	[+]	[+]	[+]
Category 5	[−]	[+]	[−]	[+]	[−]	[−]
Category 6	[−]	[+]	[+]	[+]	[−]	[−]
Category 7	[+]	[+]	[+]	[+]	[−]	[−]
Category 8	[+]	[+]	[+]	[+]	[+]	[−]
Category 9	[+]	[+]	[+]	[+]	[−]	[+]
Category 10	[+]	[+]	[+]	[+]	[+]	[+]

**Table 11:** Patterns of IgM and IgG antibodies formed against β2m and HCs of HLA-E, HLA-F, and HLA-G in different groups of RA patients during different phases of disease progression.

Groups	Tables	β2M		Heavy Chains						Phases of Disease Progression
				HLA-E		HLA-F		HLA-G		
		IgM	IgG	IgM	IgG	IgM	IgG	IgM	IgG	
Group 1	Table 2	Absent	Absent	Present/ Absent	Present/ Absent	Present/ Absent	Present/ Absent	Present/ Absent	Present/ Absent	Phase-1
Group 2	Table 3	Absent	Absent	Absent	Present/ Absent	Absent	Present/ Absent	Absent	Present/ Absent	Phase-2
Group 3	Table 4	Present	Absent	Present	Present/ Absent	Present/ Absent	Present/ Absent	Present/ Absent	Present/ Absent	Phase-3a
Group 4	Table 5	Present	Absent	Absent	Present/ Absent	Absent	Present/ Absent	Absent	Present/ Absent	Phase-3b
Group 5	Table 6	Absent	Present	Present/ Absent	Present/ Absent	Present/ Absent	Present/ Absent	Present/ Absent	Present/ Absent	Phase-3c
Group 6	Table 7	Absent	Present	Absent	Present/ Absent	Absent	Present/ Absent	Absent	Present/ Absent	Phase-3d

## Data Availability

Data are available from the first author.

## Supplementary Materials

The following supporting information can be downloaded at: https://www.mdpi.com/article/10.3390/antib12020026/s1, Table S1: Details regarding demographic data and individual treatment protocols.

## Dedication

The first author (M.H.R.) and the last author, Luis Eduardo Morales-Buenrostro, dedicate this article to our mentor, the late Paul Ichiro Terasaki, whose constant encouragements and discussions held at his office and at weekly seminars molded the thought process for this article.

## Abbreviations

ACEI: Angiotensin Convertase Enzyme inhibitor; Azat: Azathioprine; Azulf: Azulfidine; β2m: β2-Microglobulin; Chlrqn: Chloroquine; diabet: Diabetics; Dslipid: Dyslipidemia; Folic: Folic acid; HCs: Heavy Chains; HLA: Human Leukocyte Antigens; Hypertns: Hypertension; Hypothyr: Hypothyroidism; Met: Methotrexate; MFI: Mean Fluorescence Intensity; NFκB: Nuclear Factor κB; Ompr: Omeprazole; PBMC: Peripheral blood monocytes; Predns: Prednisone; RA: Rheumatoid Arthritis; Ren Dis: Renal disease; SLE: Systemic Lupus Erythematosus; Sys Vasc: Systemic Vasculitis; Thrmbss: Thrombosis.
