# Supplementary material for: Antibodies for β2-Microglobulin and the Heavy Chains of HLA-E, HLA-F, and HLA-G Reflect the HLA-Variants on Activated Immune Cells and Phases of Disease Progression in Rheumatoid Arthritis Patients under Treatment
Source: Antibodies (Basel). 2023 Mar 31;12(2):26. doi: 10.3390/antib12020026 (PMC10123671; doi:10.3390/antib12020026)
Supplement: Supplementary file 1 [file antibodies-12-00026-s001.zip › antibodies-2150459-supplementary.pdf]

Supplement Table S1. Demographic Characteristics, RA based on seropositivity to R. Factor, combinational Therapy-treatment regimens.

Total cohort	TFL CODE	MEX-ALB CODE	INSTITUTE ID	GENDER	AGE	Transfusn	number of surgeries	Pregnancy	SLE	RA	Wegner's	Sclroderms	Primary Phospholpid syndrome	Dermato myocytis	ASPL	Skin Vasculitis	Systemic Vasculitis	Connectve Tissue disease	Hypo-thyrodism	Hyper Tension	Dislipidemia	Diabetics	Secondary Phospholipid syndrome	Renal Disease	Obesity	Thrombosis	Other health Issues	Folic Acid	Azulfidine	Leflunomide	Methatrexate	Chloroquine	Azathioprine	Prednisone	Artificial tears	Omeprezole	Thyroid Enzymes	Immuno suppression	ACE Inhibitor	B-Blog	A-Blog	Calcium antoganist	Statins	NonSteroids antiinflam. Drugs
**TABLE 2**																																												
1	78	136	224,394	FEM	39	NO	1	Yes	NO	Yes	NO	NO	NO	NO	NO	NO	NO	NO	NO	NO	NO	NO	NO	NO	NO	NO	NO	NO	Yes	NO	Yes	Yes	NO	Yes	NO	NO	NO	NO	NO	NO	NO	NO	NO	NO
2	7	18	200,363	**MALE**	30	NO	0		NO	Yes	NO	NO	NO	NO	NO	NO	NO	NO	NO	NO	NO	NO	NO	NO	NO	NO	Yes	Yes	Yes	NO	Yes	NO	NO	NO	NO	NO	NO	NO	NO	NO	NO	NO	NO	Yes
3	65	121	150,764	FEM	60	NO	1	NO	NO	Yes	NO	NO	NO	NO	NO	NO	NO	NO	NO	NO	NO	NO	NO	NO	NO	NO	NO	Yes	Yes	NO	Yes	Yes	NO	NO	NO	NO	NO	NO	NO	NO	NO	NO	NO	NO
4	59	112	215,203	FEM	39	NO	2	NO	NO	Yes	NO	NO	NO	NO	NO	NO	NO	NO	NO	Yes	NO	NO	NO	NO	NO	NO	Yes	Yes	NO	NO	Yes	Yes	Yes	Yes	NO	NO	NO	NO	Yes	NO	NO	Yes	NO	NO
5	54	106	136,085	FEM	47	NO	1	Yes	NO	Yes	NO	NO	NO	NO	NO	NO	NO	NO	NO	Yes	NO	NO	NO	NO	NO	NO	NO	Yes	NO	NO	Yes	NO	NO	NO	NO	NO	NO	NO	Yes	Yes	NO	Yes	NO	NO
6	60	113	215,681	FEM	77	NO	0	NO	NO	Yes	NO	NO	NO	NO	NO	NO	NO	NO	NO	NO	NO	NO	NO	NO	NO	NO	NO	NO	NO	NO	Yes	Yes	NO	NO	NO	NO	NO	NO	NO	NO	NO	NO	NO	NO
7	26	82	171,584	FEM	51	NO	1	Yes	Yes	Yes	NO	NO	NO	NO	NO	NO	NO	NO	NO	Yes	NO	NO	NO	Yes	NO	NO	Yes	NO	NO	NO	NO	NO	Yes	NO	NO	NO	NO	NO	Yes	NO	Yes	NO	NO	NO
8	22	45	236,982	FEM	32	NO	1	Yes	NO	Yes	NO	NO	NO	NO	NO	NO	NO	NO	NO	NO	NO	NO	NO	NO	NO	NO	NO	NO	NO	NO	NO	NO	NO	NO	NO	NO	NO	NO	NO	NO	NO	NO	NO	NO
9	68	125	216,466	FEM	43	NO	0	Yes	NO	Yes	NO	NO	NO	NO	NO	NO	NO	NO	NO	NO	NO	NO	NO	NO	Yes	NO	NO	Yes	NO	NO	Yes	NO	NO	NO	NO	NO	NO	NO	NO	NO	NO	NO	NO	NO
10	17	34	158,119	FEM	48	NO	2	Yes	NO	Yes	NO	NO	NO	NO	NO	NO	NO	NO	NO	Yes	NO	NO	NO	NO	NO	NO	NO	Yes	Yes	NO	Yes	Yes	NO	NO	NO	NO	NO	NO	Yes	NO	NO	NO	NO	Yes
11	72	129	233,633	FEM	28	NO	1	NO	NO	Yes	NO	NO	NO	NO	NO	NO	NO	NO	NO	NO	NO	NO	NO	NO	NO	NO	NO	Yes	NO	NO	Yes	Yes	NO	NO	NO	NO	NO	NO	NO	NO	NO	NO	NO	NO
12	70	127	221,307	**MALE**	47	NO	1		NO	Yes	NO	NO	NO	NO	NO	NO	NO	NO	NO	NO	NO	NO	NO	NO	NO	NO	NO	Yes	NO	NO	Yes	Yes	NO	NO	NO	NO	NO	NO	NO	NO	NO	NO	NO	NO
13	44	95	209,545	FEM	64	NO	1	Yes	NO	Yes	NO	NO	NO	NO	NO	NO	NO	NO	NO	Yes	NO	Yes	NO	NO	NO	NO	Yes	Yes	NO	NO	Yes	NO	NO	NO	NO	NO	NO	NO	NO	NO	NO	NO	NO	NO
14	47	98	148,646	FEM	64	NO	4	Yes	NO	Yes	NO	NO	NO	NO	NO	NO	NO	NO	NO	Yes	NO	NO	NO	NO	NO	NO	Yes	Yes	NO	NO	Yes	Yes	NO	NO	NO	NO	NO	NO	NO	NO	NO	NO	NO	Yes
15	3	12	184,963	FEM	57	NO	4	NO	NO	Yes	NO	NO	NO	NO	NO	NO	NO	NO	NO	NO	NO	NO	NO	NO	NO	NO	Yes	NO	NO	NO	Yes	Yes	NO	NO	NO	NO	NO	NO	NO	NO	NO	NO	NO	Yes
16	10	21	170,623	FEM	65	NO	5	Yes	Yes	Yes	NO	NO	NO	NO	NO	NO	NO	NO	NO	Yes	NO	Yes	NO	NO	NO	NO	Yes	NO	Yes	NO	NO	NO	NO	NO	NO	NO	NO	NO	NO	NO	NO	NO	NO	NO
**TABLE 3**																																												
17	15	32	205,597	FEM	54	NO	1	Yes	NO	Yes	NO	NO	NO	NO	NO	NO	NO	NO	NO	NO	NO	NO	NO	NO	NO	NO	Yes	NO	NO	NO	Yes	Yes	NO	NO	NO	NO	NO	NO	NO	NO	NO	NO	NO	Yes
18	20	39	160,292	**MALE**	31	NO	0		NO	Yes	NO	NO	NO	NO	Yes	NO	NO	NO	NO	NO	NO	NO	NO	NO	NO	NO	NO	NO	Yes	NO	NO	NO	NO	NO	NO	NO	NO	NO	NO	NO	NO	NO	NO	Yes
19	14	31	201,324	**MALE**	71	NO	4		NO	Yes	NO	NO	NO	NO	NO	NO	NO	NO	NO	NO	NO	NO	NO	NO	NO	NO	Yes	NO	NO	NO	Yes	NO	NO	NO	NO	Yes	NO	NO	NO	NO	NO	NO	NO	Yes
20	55	107	86,144	FEM	65	NO	2	Yes	NO	Yes	NO	NO	NO	NO	NO	NO	NO	NO	NO	NO	NO	NO	NO	NO	NO	NO	NO	Yes	NO	NO	Yes	Yes	NO	NO	NO	NO	NO	NO	NO	NO	NO	NO	NO	Yes
21	45	96	223,952	FEM	41	NO	0	Yes	NO	Yes	NO	NO	NO	NO	NO	NO	NO	NO	NO	NO	NO	NO	NO	NO	Yes	NO	Yes	Yes	NO	NO	Yes	Yes	NO	NO	NO	NO	NO	NO	NO	NO	NO	NO	NO	Yes
22	69	126	218,813	**MALE**	26	NO	0		NO	Yes	NO	NO	NO	NO	NO	NO	NO	NO	NO	NO	NO	NO	NO	NO	NO	NO	Yes	Yes	Yes	NO	Yes	Yes	NO	NO	NO	NO	NO	NO	NO	NO	NO	NO	NO	NO
23	50	102	84,868	FEM	61	NO	3	Yes	NO	Yes	NO	NO	NO	NO	NO	NO	NO	NO	NO	Yes	NO	NO	NO	NO	NO	Yes	Yes	Yes	Yes	Yes	Yes	NO	NO	NO	NO	NO	NO	NO	NO	NO	NO	NO	NO	NO
24	43	94	187,019	**MALE**	66	NO	1		NO	Yes	NO	NO	NO	NO	NO	NO	NO	NO	NO	NO	Yes	NO	NO	NO	NO	NO	Yes	Yes	NO	NO	Yes	NO	NO	NO	NO	NO	NO	NO	NO	NO	NO	NO	NO	Yes
25	18	37	178,509	FEM	51	Yes	3	Yes	NO	Yes	NO	NO	NO	NO	NO	NO	NO	NO	NO	NO	NO	NO	NO	NO	NO	NO	Yes	Yes	NO	Yes	Yes	Yes	NO	NO	NO	NO	NO	NO	NO	NO	NO	NO	NO	NO
26	25	49	147,522	**MALE**	66	Yes	3		NO	Yes	NO	NO	NO	NO	NO	NO	Yes	NO	NO	Yes	Yes	NO	NO	Yes	Yes	NO	Yes	Yes	NO	NO	Yes	NO	NO	NO	NO	Yes	NO	NO	Yes	Yes	NO	Yes	Yes	NO
27	1	2	221,968	FEM	35	NO	2	Yes	NO	Yes	NO	NO	NO	NO	NO	NO	NO	NO	NO	NO	NO	NO	NO	NO	NO	NO	Yes	Yes	NO	Yes	Yes	NO	NO	NO	NO	NO	NO	NO	NO	NO	NO	NO	NO	Yes
28	62	115	236,748	**MALE**	40	NO	1		NO	Yes	NO	NO	NO	NO	NO	NO	NO	NO	NO	NO	NO	NO	NO	NO	NO	NO	NO	Yes	NO	NO	Yes	NO	NO	NO	NO	Yes	NO	MALE	NO	NO	NO	NO	NO	Yes
29	34	76	164,543	**MALE**	60	NO	1		NO	Yes	NO	NO	NO	NO	NO	NO	NO	NO	NO	Yes	Yes	NO	NO	NO	NO	NO	NO	Yes	NO	NO	Yes	Yes	NO	NO	NO	NO	NO	MALE	Yes	NO	NO	NO	NO	NO
30	58	111	170,296	FEM	39	Yes	0	Yes	NO	Yes	NO	NO	NO	NO	NO	NO	NO	Yes	NO	Yes	NO	NO	NO	NO	NO	NO	Yes	NO	NO	NO	Yes	Yes	NO	NO	NO	NO	NO	NO	Yes	NO	NO	NO	NO	Yes
31	28	58	194,041	**MALE**	32	NO	1		NO	Yes	NO	NO	NO	NO	NO	NO	NO	NO	NO	NO	NO	NO	NO	NO	NO	NO	NO	Yes	Yes	NO	Yes	NO	NO	Yes	NO	Yes	NO	MALE	NO	NO	NO	NO	NO	NO
32	9	20	237,638	FEM	26	NO	2	NO	NO	Yes	NO	NO	NO	NO	NO	NO	NO	NO	NO	NO	NO	NO	NO	NO	NO	NO	Yes	Yes	NO	NO	Yes	NO	NO	NO	NO	NO	NO	NO	NO	NO	NO	NO	NO	Yes
33	75	133	197,011	FEM	37	NO	2	Yes	NO	Yes	NO	NO	NO	NO	NO	NO	NO	NO	NO	NO	NO	NO	NO	NO	NO	NO	NO	Yes	NO	Yes	Yes	Yes	NO	NO	NO	NO	NO	NO	NO	NO	NO	NO	NO	Yes
34	19	38	129,471	FEM	55	NO	4	Yes	NO	Yes	NO	NO	NO	NO	NO	NO	NO	NO	Yes	Yes	NO	NO	NO	NO	Yes	NO	NO	Yes	NO	NO	Yes	Yes	NO	NO	NO	NO	NO	NO	NO	NO	NO	NO	NO	NO
35	51	103	213,477	FEM	72	NO	0	Yes	NO	Yes	NO	NO	NO	NO	NO	NO	NO	NO	NO	Yes	NO	NO	NO	NO	NO	NO	NO	Yes	NO	NO	Yes	NO	NO	NO	NO	NO	NO	NO	Yes	NO	NO	NO	NO	NO
36	64	118	237,656	FEM	30	NO	0	NO	NO	Yes	NO	NO	NO	NO	NO	NO	NO	NO	NO	NO	NO	NO	NO	NO	Yes	NO	NO	Yes	NO	NO	Yes	Yes	NO	NO	NO	Yes	NO	NO	NO	NO	NO	NO	NO	Yes
37	48	99	81,475	**MALE**	76	Yes	7		NO	Yes	NO	NO	NO	NO	NO	NO	NO	NO	NO	Yes	NO	NO	NO	NO	NO	NO	Yes	NO	NO	NO	Yes	NO	NO	NO	NO	NO	NO	MALE	Yes	NO	NO	NO	NO	NO
38	74	132	228,722	FEM	58	Yes	3	Yes	NO	Yes	NO	NO	NO	NO	NO	NO	NO	NO	NO	Yes	NO	NO	NO	NO	NO	NO	NO	Yes	NO	NO	Yes	Yes	NO	NO	NO	NO	NO	NO	Yes	NO	NO	NO	NO	Yes
39	77	135	233,833	FEM	55	Yes	1	Yes	NO	Yes	NO	NO	NO	NO	NO	NO	NO	NO	NO	NO	NO	NO	NO	NO	NO	NO	Yes	Yes	NO	NO	Yes	Yes	NO	NO	NO	Yes	NO	NO	NO	NO	NO	NO	NO	NO
40	56	109	169,519	FEM	50	NO	2	Yes	Yes	Yes	NO	NO	NO	NO	NO	NO	NO	NO	NO	Yes	NO	NO	NO	NO	NO	NO	Yes	Yes	Yes	Yes	Yes	NO	Yes	Yes	NO	NO	NO	NO	Yes	NO	NO	NO	NO	NO
**TABLE 4**																																												
41	57	110	150,003	FEM	63	NO	3	Yes	NO	Yes	NO	NO	NO	NO	NO	NO	NO	NO	NO	NO	NO	NO	NO	NO	NO	NO	Yes	Yes	NO	NO	Yes	Yes	NO	NO	NO	NO	NO	NO	NO	NO	NO	NO	NO	Yes
42	61	114	217,581	FEM	61	NO	2	NO	NO	Yes	NO	NO	NO	NO	NO	NO	NO	NO	NO	Yes	NO	NO	NO	NO	NO	NO	Yes	Yes	NO	NO	Yes	NO	NO	NO	NO	NO	NO	NO	Yes	NO	NO	NO	NO	NO
43	42	92	238,634	**MALE**	56	Yes	2		NO	Yes	NO	NO	NO	NO	NO	NO	NO	NO	NO	NO	NO	NO	NO	NO	Yes	NO	Yes	NO	NO	NO	Yes	NO	NO	Yes	NO	NO	NO	MALE	NO	NO	NO	NO	NO	NO
44	27	52	233,920	FEM	57	NO	0	Yes	NO	Yes	NO	NO	NO	NO	NO	NO	NO	NO	NO	NO	NO	NO	NO	NO	NO	NO	Yes	Yes	NO	NO	Yes	NO	NO	Yes	NO	Yes	NO	NO	NO	NO	NO	NO	NO	NO
45	26	51	231,121	FEM	44	Yes	2	NO	NO	Yes	NO	NO	NO	NO	NO	NO	NO	NO	NO	NO	NO	NO	NO	NO	NO	NO	Yes	Yes	NO	NO	Yes	Yes	NO	Yes	NO	Yes	NO	NO	NO	NO	NO	NO	NO	Yes
46	5	15	231,394	FEM	35	NO	1	Yes	NO	Yes	NO	NO	NO	NO	NO	NO	NO	NO	Yes	NO	NO	NO	NO	NO	NO	NO	Yes	NO	Yes	Yes	NO	Yes	NO	Yes	NO	Yes	NO	NO	NO	NO	NO	NO	NO	NO
47	53	105	239,112	FEM	24	NO	0	NO	NO	Yes	NO	NO	NO	NO	NO	NO	NO	NO	NO	NO	NO	NO	NO	NO	NO	NO	NO	Yes	NO	NO	Yes	NO	NO	NO	NO	NO	NO	NO	NO	NO	NO	NO	NO	Yes
48	16	33	237,047	FEM	26	NO	0	NO	NO	Yes	NO	NO	NO	NO	NO	NO	NO	NO	NO	NO	NO	NO	NO	NO	NO	NO	Yes	Yes	NO	NO	Yes	NO	NO	NO	NO	Yes	NO	NO	NO	NO	NO	NO	NO	NO
49	4	14	209,420	FEM	73	NO	0	Yes	NO	Yes	NO	NO	NO	NO	NO	NO	NO	NO	NO	Yes	NO	NO	NO	NO	NO	NO	Yes	NO	NO	NO	Yes	NO	NO	NO	NO	NO	NO	NO	Yes	NO	NO	NO	NO	Yes
50	49	100	221,223	FEM	21	NO	0	NO	NO	Yes	NO	NO	NO	NO	NO	NO	NO	NO	NO	NO	NO	NO	NO	NO	NO	NO	Yes	NO	NO	Yes	Yes	Yes	NO	NO	NO	Yes	NO	NO	NO	NO	NO	NO	NO	NO
51	8	19	237,484	FEM	25	NO	0	NO	NO	Yes	NO	NO	NO	NO	NO	NO	NO	NO	NO	NO	NO	NO	NO	NO	NO	NO	NO	NO	NO	NO	NO	Yes	NO	NO	NO	NO	NO	NO	NO	NO	NO	NO	NO	NO
52	29	60	205,159	FEM	44	NO	3	Yes	NO	Yes	NO	NO	NO	NO	NO	NO	NO	NO	NO	NO	NO	NO	NO	NO	NO	NO	NO	NO	Yes	NO	Yes	NO	NO	NO	NO	NO	NO	NO	NO	NO	NO	NO	NO	NO
53	23	47	144,043	FEM	50	NO	3	NO	NO	Yes	NO	NO	NO	NO	NO	NO	NO	NO	NO	NO	NO	NO	NO	NO	NO	NO	Yes	NO	NO	NO	NO	Yes	NO	NO	NO	NO	NO	NO	NO	NO	NO	NO	NO	Yes
54	80	138	162,807	FEM	55	NO	4	Yes	NO	Yes	NO	NO	NO	NO	NO	NO	NO	NO	NO	NO	NO	NO	NO	NO	Yes	NO	Yes	Yes	Yes	NO	Yes	NO	NO	Yes	NO	Yes	NO	NO	NO	NO	NO	NO	NO	Yes
**TABLE 5**																																												
55	38	85	199,169	FEM	47	NO	0	NO	NO	Yes	NO	NO	NO	NO	NO	NO	NO	NO	NO	Yes	Yes	Yes	NO	NO	NO	NO	NO	NO	NO	Yes	Yes	Yes	NO	NO	NO	NO	NO	NO	NO	NO	NO	NO	Yes	NO
56	24	48	145,669	FEM	41	NO	0	Yes	NO	Yes	NO	NO	NO	NO	NO	NO	NO	NO	Yes	Yes	NO	NO	NO	NO	Yes	NO	NO	NO	NO	Yes	NO	NO	NO	NO	NO	Yes	Yes	NO	Yes	Yes	NO	NO	NO	Yes
57	31	63	116,980	**MALE**	60	NO	0		NO	Yes	NO	NO	NO	NO	NO	NO	NO	NO	NO	NO	NO	NO	NO	NO	NO	NO	NO	Yes	NO	Yes	Yes	NO	NO	NO	NO	NO	NO	MALE	NO	NO	NO	NO	NO	Yes
58	67	124	216,559	FEM	29	NO	1	NO	NO	Yes	NO	NO	NO	NO	NO	NO	NO	NO	NO	NO	NO	NO	NO	NO	NO	NO	NO	Yes	Yes	NO	Yes	Yes	NO	NO	NO	NO	NO	NO	NO	NO	NO	NO	NO	NO
59	2	5	155,638	**MALE**	75	NO	2		NO	Yes	NO	NO	NO	NO	NO	NO	NO	NO	NO	Yes	NO	NO	NO	NO	NO	NO	Yes	NO	NO	NO	NO	Yes	NO	NO	NO	NO	NO	MALE	NO	NO	NO	NO	NO	Yes
60	21	43	231,731	FEM	24	NO	0	NO	NO	Yes	NO	NO	NO	NO	NO	NO	NO	NO	NO	NO	NO	NO	NO	NO	NO	NO	NO	Yes	NO	NO	Yes	Yes	NO	NO	NO	NO	NO	NO	NO	NO	NO	NO	NO	NO
**TABLE 6**																																												
61	12	27	156,223	FEM	37	Yes	7	NO	NO	Yes	NO	NO	NO	NO	NO	NO	NO	NO	NO	NO	NO	NO	NO	NO	Yes	NO	Yes	NO	NO	Yes	Yes	NO	NO	Yes	NO	NO	NO	NO	NO	NO	NO	NO	NO	Yes
62	37	84	168,713	**MALE**	62	NO	0		NO	Yes	NO	NO	NO	NO	NO	NO	NO	NO	NO	NO	NO	NO	NO	NO	NO	NO	NO	Yes	NO	NO	Yes	NO	NO	Yes	NO	NO	NO	MALE	NO	NO	NO	NO	NO	NO
63	79	137	226,218	FEM	33	NO	0	NO	NO	Yes	NO	NO	NO	NO	NO	NO	NO	NO	NO	NO	NO	NO	NO	NO	NO	NO	Yes	Yes	Yes	NO	Yes	Yes	NO	NO	NO	NO	NO	NO	NO	NO	NO	NO	NO	Yes
64	76	134	211,699	FEM	45	NO	0	Yes	NO	Yes	NO	NO	NO	NO	NO	NO	NO	NO	NO	NO	NO	NO	NO	NO	NO	NO	NO	Yes	NO	NO	Yes	Yes	NO	NO	NO	Yes	NO	NO	NO	NO	NO	NO	NO	Yes
65	39	88	164,563	FEM	34	NO	3	Yes	NO	Yes	NO	NO	NO	NO	NO	NO	NO	NO	NO	NO	NO	Yes	NO	NO	NO	NO	Yes	NO	NO	NO	Yes	NO	NO	Yes	NO	NO	NO	NO	NO	NO	NO	NO	NO	Yes
**TABLE 7**																																												
66	40	90	203,257	FEM	41	NO	1	Yes	NO	Yes	NO	NO	NO	NO	NO	NO	NO	NO	NO	NO	NO	NO	NO	NO	NO	NO	NO	NO	NO	NO	Yes	Yes	NO	NO	NO	NO	NO	NO	NO	NO	NO	NO	NO	NO
67	13	30	67,081	**MALE**	58	Yes	11		NO	Yes	NO	NO	NO	NO	NO	NO	NO	NO	NO	NO	NO	NO	NO	NO	NO	NO	Yes	NO	NO	NO	NO	NO	NO	NO	NO	NO	NO	MALE	NO	NO	NO	NO	NO	Yes
68	46	97	227,459	FEM	47	NO	0	Yes	NO	Yes	NO	NO	NO	NO	NO	NO	NO	NO	NO	NO	NO	NO	NO	NO	NO	NO	Yes	Yes	NO	NO	Yes	Yes	NO	Yes	NO	NO	NO	NO	NO	NO	NO	NO	NO	NO
**TABLE 8**																																												
69	41	91	213,683	FEM	30	NO	0	NO	NO	Yes	NO	NO	NO	NO	NO	NO	NO	NO	NO	NO	NO	NO	NO	NO	NO	NO	Yes	NO	NO	NO	Yes	NO	NO	NO	NO	NO	NO	NO	NO	NO	NO	NO	NO	Yes
70	11	24	216,070	FEM	54	NO	4	Yes	NO	Yes	NO	NO	NO	NO	NO	NO	NO	NO	Yes	NO	NO	NO	NO	NO	NO	NO	Yes	NO	NO	NO	Yes	NO	NO	NO	NO	NO	Yes	NO	NO	NO	NO	NO	NO	Yes
71	32	65	212,184	FEM	59	NO	1	Yes	NO	Yes	NO	NO	NO	NO	NO	NO	NO	NO	NO	Yes	NO	Yes	NO	NO	NO	NO	Yes	Yes	NO	NO	Yes	Yes	NO	NO	NO	NO	NO	NO	NO	NO	NO	NO	NO	Yes
72	52	104	220,898	FEM	22	NO	0	NO	NO	Yes	NO	NO	NO	NO	NO	NO	NO	NO	NO	NO	NO	NO	NO	NO	NO	NO	Yes	NO	NO	NO	Yes	Yes	NO	Yes	NO	NO	NO	NO	NO	NO	NO	NO	NO	NO
73	73	130	215,930	FEM	51	NO	0	Yes	NO	Yes	NO	NO	NO	NO	NO	NO	NO	NO	NO	NO	NO	NO	NO	NO	NO	NO	NO	Yes	NO	NO	Yes	Yes	NO	NO	NO	Yes	NO	NO	NO	NO	NO	NO	NO	NO
74	33	74	226,016	FEM	37	NO	2	NO	NO	Yes	NO	NO	NO	NO	NO	NO	NO	NO	NO	NO	NO	NO	NO	NO	NO	NO	Yes	Yes	Yes	NO	Yes	Yes	NO	NO	NO	Yes	NO	NO	NO	NO	NO	NO	NO	Yes
